# Combined photodynamic therapy and intravitreal ranibizumab as treatment for extrafoveal choroidal neovascularization associated with age-related macular degeneration

**DOI:** 10.4103/0974-620X.64241

**Published:** 2010

**Authors:** Jay Kumar Chhablani

**Affiliations:** Smt. Kanuri Santhamma Retina Vitreous Centre, L V Prasad Eye Institute, Hyderabad, India

Sir,

We report the efficacy of combination therapy using photodynamic therapy (PDT) and intravitreal ranibizumab for extrafoveal choroidal neovascular membrane (CNVM) associated with age-related macular degeneration (ARMD) in an Indian patient.

**Figure 1:**
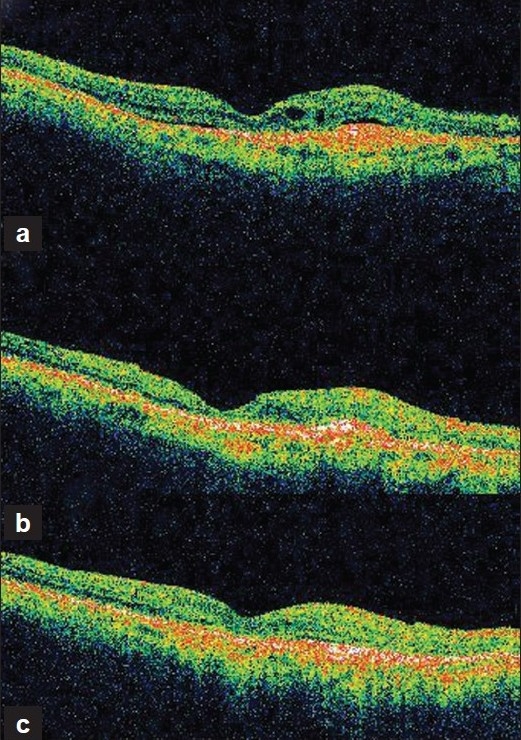
Serial Optical Coherence Tomography (OCT) (a-c) showing decrease in retinal thickness, resolution of subretinal fluid with restoration of normal foveal contour

A 74-year-old man presented with decreased vision in the right eye (OD) since two years. He was a known diabetic on treatment. He was on anti-glaucoma medication for both the eyes (OU). On examination his best corrected visual acuity was 20/80 OD and 20/2000 in the left eye (OS). Biomicroscopic evaluation of the anterior segment of both the eyes was unremarkable except for aphakia. Intraocular pressures were 12 mmHg OU. Fundus examination OD revealed a subretinal membrane with retinal pigment epithelium alteration. The optic disc showed signs of glaucomatous cupping (cup-disc ratio 0.6). Fundoscopy OS showed macular scarring with glaucomatous cupping (cup-disc ratio 0.6). Fundus fluorescein angiography (FFA) showed typical lacy leakage suggestive of classic CNVM OD. Optical coherence tomography (OCT) showed retinal thickening with subretinal fluid [Figure 1a]. Considering the location of the CNVM and the single-eyed status of the patient, laser photocoagulation was thought to be best avoided. The patient underwent PDT (5.3 ml of Verteporfin in 24.3 ml of D5W, laser spot size of 2.6mm, using diode laser (wavelength 689nm) as per standard protocol followed by intravitreal ranibizumab (0.5 mg), two days later in the right eye. No treatment-related adverse effect was noted. At four weeks follow-up, visual acuity improved to 20/40. Clinical examination revealed absence of subretinal fluid but persistence of increased retinal thickness on OCT [Figure 1b]. A second intravitreal ranibizumab injection was performed. At eight weeks follow-up, visual acuity improved to 20/30. Clinical examination revealed a scarred CNVM [Figure 1c]. The fundus remained stable and visual acuity was maintained at the sixth month follow-up.

Combined treatment using PDT and bevacizumab has been shown to be effective in improving visual acuity and decreasing retreatment rates in choroidal neovascularization (CNV) associated with ARMD.[1] The combined regime is postulated to have a beneficial synergistic effect that reduces the need for cyclic injections.[1,2]

Heier *et al*. reported greater efficacy with combination therapy using ranibizumab and PDT when compared to therapy with PDT alone.[3] VEGF inhibition alone could prevent neovascularization at an early developmental stage. However, once neovascular beds are established they are unlikely to regress with anti-VEGF therapy alone.[3]

Combination therapy treatment using PDT and ranibizumab as the first-line management in treatment of CNVM in one-eyed patients is a safe and effective treatment option, leading to a permanent cure. Combination therapy has a beneficial effect of reduced number of injections on reducing the attendant cumulative risk of endophthalmitis with each additional injection.
